# Potentials of post-mortem CT investigations during SARS-COV-2 pandemic: a narrative review

**DOI:** 10.1007/s11547-022-01457-w

**Published:** 2022-02-28

**Authors:** Laura Filograna, Guglielmo Manenti, Garyfalia Ampanozi, Antonello Calcagni, Colleen Patricia Ryan, Roberto Floris, Michael John Thali

**Affiliations:** 1 Department of Radiological Sciences, Fondazione PTV Policlinico Tor Vergata, Viale Oxford 81, 00133 Rome, Lazio, IT Italy; 2 Institute of Forensic Medicine, Department of Forensic Medicine and Imaging, University of Zurich, Winterthurerstrasse 190/52, CH-8057 Zurich, Switzerland

**Keywords:** Virtual autopsy, Infection risk reduction, Sars-Cov-2, Post-mortem CT, COVID-19 CT findings

## Abstract

In December 2019, a new coronavirus, SARS-COV-2, caused a cluster of cases of pneumonia in China, and rapidly spread across the globe. It was declared a pandemic by the World Health Organization on March 11th, 2020. Virtual autopsy by post-mortem CT (PMCT) and its ancillary techniques are currently applied in post-mortem examinations as minimally or non-invasive techniques with promising results. In this narrative review, we speculate on the potentials of PMCT and its ancillary techniques, as a viable investigation technique for analysis of suspected or confirmed SARS-COV-2 deaths. An online literature search was performed by using three prefix search terms (postmortem, post-mortem, post mortem) individually combined with the suffix radiology, imaging, computed tomography, CT and with the search terms ‘SARS-CoV-2’ and ‘COVID-19’ to identify papers about PMCT and its ancillary techniques in SARS-COV-2 positive cadavers. PMCT findings suggestive for pulmonary COVID-19 in deceased positive SARS-COV-2 infection are reported in the literature. PMCT ancillary techniques were never applied in such cases. PMCT imaging of the lungs has been proposed as a pre-autopsy screening method for SARS-COV-2 infection. Further studies are needed to ascertain the value of PMCT in determining COVID-19 as the cause of death without autopsy histopathological confirmation. We advocate the application of PMCT techniques in the study of ascertained or suspected SARS-COV-2 infected deceased individuals as a screening technique and as a method of post-mortem investigation, to augment the numbers of case examined and significantly reducing infection risk for the operators.

## Introduction

In December 2019, a new coronavirus was the cause of a cluster of cases of pneumonia that originated in China, and subsequently spread across the world. It was declared a pandemic by the World Health Organization (WHO) on March 11th, 2020 [1]. The coronavirus family comprises several viruses that cause serious human diseases including Middle East respiratory syndrome (MERS) and severe acute respiratory syndrome (SARS) [2]. The SARS-coronavirus-2 (SARS-COV-2) causes an illness in humans named as coronavirus disease 2019 (COVID-19) by WHO [1, 3].

Although the upper respiratory tract and lungs represent the predominant site of entry and replication of SARS-COV-2, other replication sites such as endothelial cells and the kidney have been suggested [4, 5].

The major manifestations of COVID-19 involve the respiratory tract; however, extrapulmonary manifestations of COVID-19 infection, particularly neurological manifestations, have been well described in the literature [6, 7].

Until now, several studies based on autopsy cases have been published about the histopathological findings in SARS-COV-2 infection [8–11]. Autopsy or other alternative post-mortem investigations are essential to elucidate pathophysiological mechanisms of SARS-COV-2 and contributed enormously to advances in the prevention and treatment of patients with COVID-19.

In these scenarios, while performing complete autopsy is of outmost importance, the contamination risk of infection for operators during body sectioning is a critical issue. Despite recommendations from some authors and scientific societies, such as the Italian Society of Anatomical Pathology (SIAPEC), to restrict autopsies to selected cases at a first time, many forensic institutions have continued to regularly perform autopsies since the beginning of the pandemic [12, 13]. However, if death occurred in a person with suspected or confirmed SARS-COV-2 infection, but is involved in forensic investigation, performing an autopsy is necessary in most cases. The roles, indications and guidelines for the correct management of potential infectious pathogens applied in clinical and research practice in microbiology laboratories have been translated to mortuary setting to reduce transmission risk of these pathogens during and after post-mortem examinations.

In the deceased where SARS–COV-2 is suspected or ascertained, as well as for other high risk of infection pathogens, some guidelines for safe autopsy procedures have been defined in Italy by the Italian National Institute of Health (INIH) [14].

Essentially, the precautions indicated by INIH and other international health organizations [15, 16] are related to the environment where the autopsy is performed, protection of the operator, selection of the operator and knowledge of the case history.

Thus, because autopsies performed on people with high risk of infection request specific procedures and environmental conditions in the autopsy room, high numbers of autopsies on SARS-COV-2 cases were difficult to achieve, at least during the first peak of the pandemic.

The term “Virtual autopsy” or “Virtopsy®” was defined in 2002 by Prof. Thali et al. at the Institute of Forensic Medicine of the University of Bern, Switzerland, as the set of imaging techniques used on a cadaver for forensic purposes [17, 18]. The fundamental basis for a modern virtual autopsy is undoubtedly a whole-body post-mortem CT (PMCT) scan in high-resolution due to the ability to neglect radiation exposure and the absence of motion artifacts [19, 20]. Although PMCT is considered very useful to analyze violent deaths due to the optimal visualization of skeletal lesions, intravascular gas, foreign bodies, this technique shows limitations in investigating "natural" deaths, due to its poor ability in differentiating soft tissue interfaces and in documenting vascular alterations [17]. In 2005, PMCT angiography, firstly introduced by Jackowski et al. [21] and after improved by variating the technique and the type of intravascular contrast media [22–24], overcame most limitations in documenting vascular alterations.

However, a significant limit of PMCT remained the absence of body samples for histopathological, microbiological or toxicological analysis. To overcome this limit, by 2007, post-mortem percutaneous biopsy [25–27] proved to successively provide specimens suitable for histological examination.

Since then, post-mortem imaging techniques, primarily PMCT, have also been successfully applied to the investigation of non-forensic cases. In fact, these post-mortem modalities have been used for pathological investigations and even in veterinary settings [28–30].

In this narrative review, we speculate on the potentials of the modern techniques of virtual autopsy by PMCT and its ancillary techniques, mainly PMCT angiography and percutaneous biopsy, as a viable non-invasive, or minimally invasive technique for analysis of suspected or confirmed SARS-COV-2 deaths, to favor the reduction of operator risk of infection and to increase the availability of post-mortem data, on a histopathological level in individuals who died with SARS-COV-2 infection. PMCT findings in deceased positive SARS-COV-2 infection, especially focusing on the lungs, as reported in the current available literature are also presented. Finally, possible applications of ancillary to PMCT techniques in post-mortem investigations on SARS-COV-2 cases are also discussed. With the aim to cover the entire pertinent literature, one of the authors, a board-certified radiologist and forensic pathologist with more than fifteen years of experience in post-mortem imaging, independently searched in two databases, PubMed and Google Scholar. Three prefix search terms (postmortem, post-mortem, post mortem) were individually combined with the suffix radiology, imaging, computed tomography, CT and with the search terms ‘SARS-CoV-2’ and ‘COVID-19’. The inclusion criteria were all types of articles and related only to humans. The exclusion criteria were articles for which full text was not available, were not in English. From the articles retrieved in the first round of search, additional references were examined by a manual search among the cited references.

## CT findings in living patients

It is well demonstrated that SARS-COV-2 affects principally the lungs. Based on the available biopsy or autopsy studies, pulmonary pathology [11, 31] in SARS-COV 2 infection is represented by diffuse alveolar damage due to the development of hyaline membranes, macrophage activation in the air spaces, and thickening of the alveolar wall.

Many clinical studies have reported different CT patterns of pulmonary involvement [32–39]. According to the recent literature [38, 39], the most typical of chest CT findings in COVID-19 patients are bilateral lung involvement by ground-glass opacity (GGO) or mixed GGO and consolidation, thickened interlobular septa, crazy-paving pattern, vascular enlargement, air bronchogram sign, peripheral distribution, and left and right lower lobes involvement. However, the same pattern identified as typical for COVID-19 in the lungs, is common with the lung CT pattern caused by other viral pathogens, for example virus such as H7N9, H1N1 virus infection, SARS, MERS, and avian influenza A (H5N1) [40–43], or other sources of lung pathology such as drug reactions. Nevertheless, chest CT scan is considered an important part of disease diagnosis for patients suspected of having COVID-19, especially those with negative initial reverse-transcription polymerase chain reaction (RT-PCR) screening [44]. In fact, given the limited number of RT-PCR kits in many centers and the likelihood of false negative RT-PCR results, the National Health Commission of the People's Republic of China, has suggested CT as a major modality for diagnosis, even before receiving the RT-PCR tests [45].

Other organs are affected by SARS-COV-2 due to direct attack by the virus or through an indirect mechanism mediated by inflammation and coagulation mediators [46, 47]. The lymph nodes, heart, kidneys, spleen, liver, gastrointestinal tract and brain have all been indicated as organs affected by multiorgan dysfunction in COVID-19 disease. Some studies report CT findings in extrapulmonary manifestations of COVID-19 [46], mainly focusing on the brain and the bowel. In the brain, due to potential endothelial damage, SARS-COV-2 may cause hemorrhagic lesions [48, 49]. Possible CT manifestations of SARS-COV-2 involvement of the brain are hypodensity within the bilateral medial thalami on the head CT and hemorrhagic lesions [50, 51]. Regarding the gastrointestinal tract, non-specific findings such as distended fluid filled small and large bowel loops and surrounding stranding, intestinal wall thickening on clinical non-enhanced CT have been reported, as the expression of parietal bowel damage in COVID-19 [46, 52–55]. Uncommonly, mediastinal lymphadenopathy with lymph node enlargement potentially detected by CT imaging has been reported in the literature in critically ill COVID-19 patients, as an expression of the inflammatory response to the infection [56]. SARS-COV-2 was also detected in the liver cells and microvesicular steatosis of moderate entity and mild lobular and portal inflammatory activity were reported in some histopathological studies on SARS-COV-2 patients [46]. The literature suggests that 71,4% of non-survivors of COVID-19 suffered from disseminated intravascular coagulation, with abnormal coagulation results in later stages of the disease [57]. In critically ill COVID-19 patients two types of pathologic coagulation processes can be identified, one involving the microcirculation of the lungs and other organs causing microvascular clots [58], and the other affecting the systemic circulation with the potential development of large vessel thrombosis and major thromboembolic events, including pulmonary embolism [59–61].

## Potential use of postmortem CT in SARS-COV-2 positive deaths

The literature research of this narrative review retrieved papers regarding the use of PMCT in SARS-COV-2 positive cadavers [62–70]. It is interesting to note that all the selected papers were focused on PMCT appearances of SARS-COV-2 lung disease. Papers about extrapulmonary manifestations of COVID-19 on PMCT or about ancillary PMCT techniques (i.e., PMCT angiography and PMCT guided percutaneous biopsy) applied in SARS-COV-2 positive cadavers were not published.

### PMCT of the lungs

The potentials of PMCT in identifying significant pulmonary alterations in post-mortem investigations are well known [71–73]. The presence of air in the lungs provides an intrinsic contrast, that renders the lungs one of the organs best imaged on non-enhanced CT in clinical and post-mortem setting. PMCT imaging of the lungs is considered a valuable tool in forensic investigations, and it is employed as a post-mortem method of analysis as an adjunctive or even substitutive tool for autopsy [74]. As well as in other organs, in the pulmonary parenchyma the formation of internal livors affects their PMCT imaging, even more than in other organs. Internal hypostases in the lungs appear as an attenuation gradient with areas of grater opacity mainly of ground glass type, localized in dependent regions of the lungs (e.g., positions that may vary depending on the decubitus after death) [71].

These normal post-mortem alterations on PMCT imaging might mask an underlying COVID-19 lung involvement. This is theoretically more probable in initial stages of lung pathology, when few and scanty areas of ground glass opacity could be in dorsal, peripheral regions, eventually in the inferior lobes, thus with the same distribution of internal livors. If we do not take in to account the increase of the risk of infection, forced mechanical ventilation of cadaveric lungs might be proposed to improve the detection of COVID-19 related lung alterations. In fact, this technique has been proved to be a valuable method to reduce PMCT findings related to hypostases in the lungs and to enhance relevant pathological pulmonary PMCT findings [75].

**Fig. 1:**
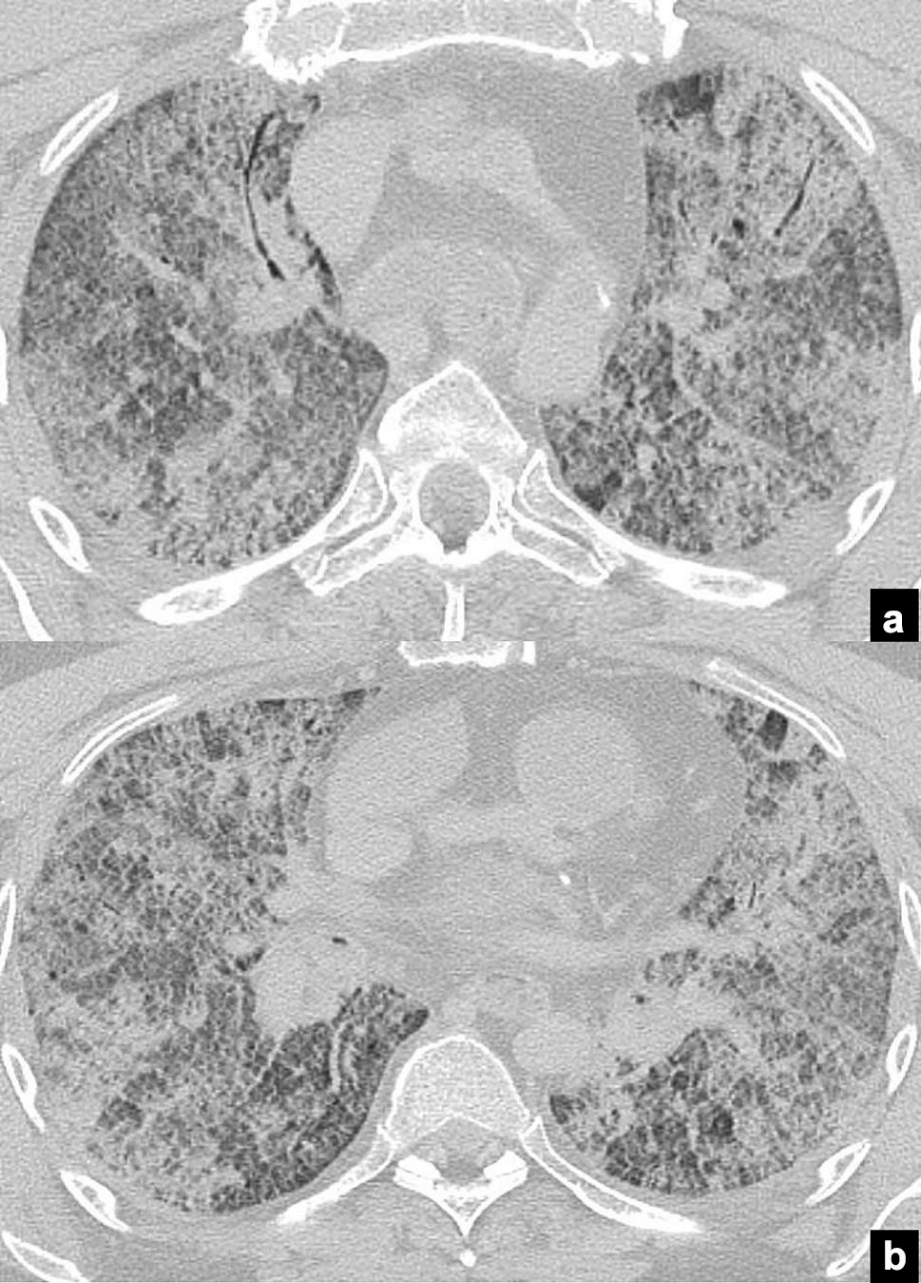
Case example of advanced COVID-19 pulmonary involvement on PMCT imaging. Axial PMCT images at the level of the thorax in a deceased with ascertained SARS-COV-2 infection. PMCT images (**a**, **b**) show bilateral massive involvement of pulmonary parenchyma with diffuse crazy paving pattern and multiple areas of consolidations, with some air bronchogram (**b**)

The presented review of the literature confirmed the value of PMCT in cadavers positive for SARS-COV-2 infection. The most common findings on PMCT reported in the literature as the expression of severe pulmonary COVID-19, as it occurs in clinical setting, were bilateral, mixed pattern of GGOs, commonly with crazy paving pattern, and consolidations, in either a diffuse or a peripheral distribution [62–70] (Fig. 1 exemplar). An adjunctive, even more not specific pattern was diffuse consolidation and GGO of both lungs [67, 69, 70], described by O’Donnell as “dense airless lungs”, attributed to advanced COVID-19 pneumonitis in clinical setting [32–39], can be interpreted as a common final common pathway in respiratory illness related to severe lung involvement by the pathogen, resembling adult respiratory distress syndrome (ARDS).

In the previous publications about PMCT alterations of the lungs in SARS-COV-2 positive cases, this post-mortem investigation tool was basically proposed as a screening method for SARS-COV-2 infection in cadavers [62–68, 70]. Regarding the possibility to attribute the cause of death based on PMCT imaging, it was most commonly [68–70] retained that there is currently insufficient published literature and the findings reported for assessment of SARS-CoV-2 infection of the lungs using PMCT are not sufficiently specific to propose this post-mortem imaging technology as an alternative to autopsy and histopathological, and microbiological analysis.

### PMCT of other organs

As previously mentioned, other organs are affected by SARS-COV-2 due to direct attack or by indirect mechanisms [6, 46, 47].

Although no PMCT imaging findings corresponding to these pathological alterations have yet been reported, most of the ante-mortem extrapulmonary CT alterations suggested in the literature as related to SARS-COV-2 infection, might be theoretically identified in PMCT imaging. Namely, in the brain as well as in clinical CT, the endothelial damage caused by SARS-COV-2 infection might be expressed by ischemic or hemorrhagic lesions [48–51].

Other possible extrapulmonary CT findings may be mediastinal lymphadenopathy with lymph node enlargement [56], large bowel loops and surrounding stranding, intestinal wall thickening [46, 52–55], diffuse hypodensity of the liver due to non-specific steatosis [46]. Regarding the CT imaging findings related to disseminated intravascular coagulation elicited by SARS-COV-2 in critically ill patients [57–61], it is of interest to note that unenhanced PMCT has been used to document central pulmonary embolism based on the shape of the vascular content within the pulmonary trunk and arteries [76, 77]. Moreover, it has been verified that distension of the inferior vena cava on PMCT suggests the presence of pulmonary thromboembolism with a specificity of 83% and a sensitivity of 54% [76].

### PMCT angiography

PMCT angiography has been introduced in virtual autopsy as a complementary technique; this technique adds crucial information about vascular bed in non-decomposed cadavers, enhancing virtual autopsy, particularly in natural deaths [21–24, 78]. Some studies have demonstrated the capability of PMCT angiography to document central and paracentral pulmonary thromboembolism [22, 79], and a complete concordance with autopsy results and histological analysis was verified. Also, deep venous thrombosis has been demonstrated in some cases of PMCT angiography with known pulmonary embolism [79]. Otherwise, the development of small post-mortem clots and/or the presence of possible minute filling defects due to artifacts generally impairs the correct identification of more peripheral pulmonary embolism [22].

Thus, in cases of suspected lethal pulmonary embolism, the diagnostic accuracy of PMCT angiography in diagnosing pulmonary embolism is not completely proven, due to the absence of large cadaveric studies. Furthermore, post-mortem clots and artifacts may compromise the accuracy of this post-mortem imaging technique in demonstrating pulmonary embolism. In any case, PMCT angiography can be suggested as a viable technique for detecting central and paracentral pulmonary embolism or other major thrombotic or thromboembolic phenomena in a minimally invasive manner in deceased individuals with COVID-19. Nevertheless, the reduced ability of PMCT angiography to demonstrate microvascular thrombosis and embolism renders the histological analysis more desirable.

### PMCT-guided biopsy

In the setting of minimally invasive autopsy, post-mortem multiorgan percutaneous biopsies have already been successfully applied to post-mortem investigations of three cases of deceased COVID-19 patients [80]. Post-mortem percutaneous biopsies with PMCT guidance may represent a useful technique for focused minimally invasive post-mortem investigations in deceased individuals with ascertained or suspected SARS-COV-2 infection. In this scenario, post-mortem percutaneous biopsies may permit to overcome the above-mentioned limitations of virtual autopsy with unenhanced PMCT or also with PMCT angiography, by providing tissue and body fluid samples for histological, immunohistochemical, microbiological and toxicological analysis.

## Advantages of PMCT in SARS-COV-2 infected deaths

### SARS-COV 2 infection is the cause of severe disease with a rapid pandemic diffusion

In the post-mortem setting, Italian National Institute of Health (INIH) has underlined the importance of histopathological, microbiological and virologic analysis on tissue and biological specimens of deceased individuals with ascertained and suspected SARS-COV-2 infection. The aim for autopsy procedure on ascertained cases of COVID-19 is mainly to help in understanding pathophysiological mechanisms of SARS-COV-2 which in turn may contribute enormously to advances in prevention and treatment of patients with COVID-19. On the other hand, the contamination risk for operators during body sectioning is equally a crucial issue, and INIH and other health institutions worldwide have advocated the limitation of body dissections in ascertained and, particularly, in suspected SARS-COV-2 deceased patients. Moreover, due to high mortality rates in many regions of Italy and worldwide, especially in the first peak of pandemic, the large number of deceased individuals to be examined in addition to the proposition to reduce infective risk for health operators undoubtedly limits the rates of autopsy on SARS-COV-2 infected individuals. Furthermore, during the first peak of pandemic, every cadaver to be dissected, also for forensic purposes, must be considered as potentially infected and must undergo post-mortem management of potentially high grade HG3 pathogens.

Other limits to full autopsy are the availability of adequate mortuary rooms, protective devices, and trained operators to guarantee acceptable reduction of risk of transmission of infection to third parties. The same INIH in the document reporting indications about autopsy procedures [14] states “in patients dying with SARS-CoV-2 infection, the autopsies can confirm laboratory and radiological findings […]”.

Although in a recent systematic review about the development of forensic imaging in Italy an increase of the use of these techniques, particularly PMCT, has been observed in the last years, the literature demonstrated that imaging techniques are not yet exhaustively adopted by the Italian forensic community, and that most publications are from authors affiliated with university groups of research [81]. However, due to the wide diffusion of CT in the Italian territory both in university and in extra-university context, we are convinced that PMCT can represent the technique of choice for post-mortem investigations in pandemic emergencies.

With this contribution, we suggest the use of these post-mortem investigation techniques in cadavers of ascertained and suspected SARS-COV-2 infected individuals.

The advantages are several including that these procedures, minimally or non-invasive, can significantly reduce the risk of infection of the operators. Above all, if exclusively a whole body PMCT is performed, preliminary information about a potential COVID-19 lung disease is achievable, particularly in cadavers where clinical information before death is not available and the complete results of viral nose and throat swab are still not available. Moreover, a whole body PMCT is rapid and does not require specific environmental conditions, different to those normally applicable on living patients. Thus, the numbers of cadavers undergoing post-mortem analysis could be significantly increased.

Moreover, in ascertained cases of death due to COVID-19 focused, eventually CT guided, post-mortem percutaneous biopsies may improve the understanding of this lethal pathology with important impact on prevention and treatment, but with consistent reduction of infection for the operators unlike in classical autopsy techniques.

Finally, a general advantage of this post-mortem technique is to provide an objective data set available for further analysis.

## Conclusions

For all the aforementioned reasons, we advocate the application of PMCT virtual autopsy techniques in the study of ascertained or suspected SARS-COV-2 infected deceased individuals as a screening technique and as a method of post-mortem investigation. These techniques might be able to provide significant information about COVID-19 on many deceased patients while significantly reducing infection risk for the operators.
